# Benchmarking Mechanical Properties of 3D Printed Elastomeric Microstructures

**DOI:** 10.1002/smtd.202500432

**Published:** 2025-06-12

**Authors:** Or Eivgi, Clara Vazquez‐Martel, Jaroslav Lukeš, Eva Blasco

**Affiliations:** ^1^ Institute for Molecular Systems Engineering and Adv. Mater. (IMSEAM) Heidelberg University Im Neuenheimer Feld 225 69120 Heidelberg Germany; ^2^ Bruker Nano Surfaces and Metrology Technická 4 Prague 160 00 Prague 6 Czech Republic

**Keywords:** additive manufacturing, elastomers, mechanical properties, nanoindentation, PDMS, two‐photon polymerization

## Abstract

The characterization of mechanical properties in soft three‐dimensional (3D) printed materials at the microscale remains a significant challenge due to the lack of standardized methodologies. To address this issue, a microscale nanoindentation protocol for elastomeric 3D printed microstructures is developed, optimized, and benchmarked. Herein, a conospherical indenter tip (r = 10.26 µm), a modified trapezoidal displacement profile with lift‐off segments to capture adhesion interactions, and the nano‐Johnson–Kendall–Roberts model for data analysis are employed. The protocol is optimized and verified using four newly developed polydimethylsiloxane (PDMS)‐based inks for two‐photon 3D laser printing. The results are compared to a state‐of‐the‐art literature protocol that uses a Berkovich tip and the Oliver–Pharr model. It is shown that adhesion forces play a significant role in mechanical properties overestimation, showing differences of up to 80% between the different protocols. This study highlights the importance of carefully selecting characterization protocol to yield comparable results between studies. By providing a standardized protocol, it paves the way for straightforward and accurate characterization of mechanical properties in soft 3D printed materials at the microscale.

## Graphical abstract

A nanoindentation protocol for three‐dimensional (3D) printed elastomeric microstructures is developed, optimized, and benchmarked to address tip‐sample adhesion and substrate artifacts. Elastic modulus measurements are shown to be strongly influenced by sample geometry, test parameters, indenter tip shape, and analysis models. The importance of standardized characterization protocols is highlighted to ensure accurate and comparable microscale mechanical data for soft 3D‐printed materials.

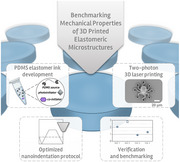

## 1 Introduction

Elastomers are a class of polymeric materials known for their viscoelasticity, low stiffness and high failure strain.^[^ ^1^ ^]^ In recent years, with the evolution of 3D printing and additive manufacturing technologies, macro‐ and microscale 3D printing of elastomers has drawn significant attention in academia and industrial settings.^[^ ^2^ ^]^ 3D printing of mechanically soft materials has found a wide range of applications across industries from automotive and wearable electronics to healthcare and soft robotics.^[^ ^3^ ^]^ For all of these applications, the mechanical characterization of the 3D printed elastomers is fundamental for understanding their interaction with the environment and is a key element in establishing structure‐property relationships.^[^ ^4^ ^]^ While mechanical characterization of rigid materials such as metals is relatively straightforward and standardized measurement methods exist, the evaluation of mechanical properties of 3D printed elastomers presents unique challenges that increase as the size of the 3D printed structures decreases. First, almost all classes of materials exhibit a so‐called “size effect”, where their properties vary when scaled down to micrometer and nanometer size, making it unreliable to extrapolate measurements obtained with macroscopic samples.^[^ ^5^, ^6^ ^]^ Furthermore, the characterization of mechanical properties on the microscale requires specialized^[^ ^7^ ^]^ and often expensive microscopy‐based equipment with high spatial resolution such as microscale compression setups, atomic force microscopy (AFM), optical tweezers or by using nanoindentation.^[^ ^8^, ^9^ ^]^

Nanoindentation is a microscale contact mechanics method that uses a hard indenter probe tip with known and well‐characterized mechanical properties (often made of diamond) to deform a sample surface of unknown properties.^[^ ^10^ ^]^ It has gained popularity in the last two decades for characterizing mechanical properties at the microscale for rigid materials,^[^ ^11^, ^12^, ^13^ ^]^ but also for soft and biological samples, due to its ability to measure samples with limited dimensions and its sensitivity to sample anisotropy and inhomogeneity.^[^ ^9^ ^]^ In their tutorial review article, Pruitt and co‐workers provide guidelines on performing nanoindentation measurements for soft materials and suggest the use of a conospherical tip, displacement‐controlled method and a modified trapezoidal function to probe compliant, adhesive materials.^[^ ^14^ ^]^ This approach was validated by Gallant and co‐workers for macro‐scale polydimethylsiloxane (PDMS) samples.^[^ ^15^ ^]^

However, as of today, there are only few reports on the characterization of mechanical properties of microscale 3D printed soft elastomers using nanoindentation, with a lack of clear methodology. Accardo and co‐workers studied mechanical properties of 3D printed PDMS microstructures (IP‐PDMS, Nanoscribe GmbH & Co. KG) using nanoindentation with a conospherical tip. By varying the printing parameters, a wide range of stiffness varying between 0.35 and 17.8 MPa was reported, although adhesion effects were not taken into consideration.^[^ ^16^ ^]^ The lack of standardization for performing nanoindentation on microscopically patterned elastomeric materials increases the risk of variability and potentially reporting erroneous results, especially as measurements become more sensitive to experimental conditions and equipment parameters.^[^ ^14^ ^]^ Since elastomeric microprinted structures, and in particular PDMS‐based inks, are used for cellular biology experiments, obtaining accurate mechanical properties characterization data of such microscaffolds is of essence.^[^ ^6^, ^17^ ^]^

**Figure 1:**
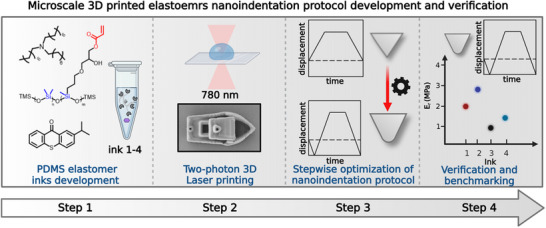
Protocol development workflow – PDMS based inks development, two‐photon 3D laser printing, stepwise optimization of the nanoindentation protocol and verification of the protocol using 3D printed samples from the inks prepared.

Herein, we report a standardized nanoindentation protocol for benchmarking of mechanical properties characterization of 3D printed elastomers on the microscale (**Figure** **1**). This protocol is developed following a stepwise optimization of the different testing parameters (indentation tip geometry, displacement profile, peak indentation depth and selecting a suitable model for data analysis and extraction of the mechanical properties). The standardization and benchmarking were performed by thoroughly evaluating the mechanical properties of soft elastomeric microstructures printed by two‐photon 3D laser printing (2PLP) using four different PDMS‐based elastomer inks, developed for this purpose. This new protocol uses a small‐diameter conospherical tip, that allows the high spatial resolution needed for 3D printed microscale objects. In order to record adhesion effects during approach and withdrawal from the sample, modified displacement‐controlled displacement profiles including lift‐off segments at the start and end of the indent were employed. Moreover, appropriate contact models, such as the nano‐Johnson–Kendall–Roberts (nano‐JKR) model, have been employed to analyze the nanoindentation data.^[^ ^18^, ^19^ ^]^ The mechanical properties obtained using the proposed protocol were compared to a state‐of‐the‐art literature reported nanoindentation protocol using a Berkovich tip and the Oliver–Pharr (OP) model.^[^ ^11^, ^20^, ^21^, ^22^ ^]^ The results show significant differences in the mechanical properties reported for 3D printed soft elastomeric materials, and point out the significance of the standardized protocol proposed.

## 2 Results and Discussion

### 2.1 Printability Evaluation of Commercial Acrylated PDMS Sources and PDMS Elastomer Inks Preparation

In order to accurately assess a 3D printed soft elastomers nanoindentation protocol the development of a set of soft PDMS inks for 2PLP with a stiffness gradient was envisioned. This is also due to the fact that the choice of commercially available soft‐elastomer inks suitable for 2PLP is limited.

To ensure accessibility to a broad scientific community, commercially available acrylated PDMS products were chosen as the core of the new soft elastomer inks. However, when it comes to commercial PDMS products, it is often difficult to obtain exact data on its composition, making it difficult to evaluate the suitability of the acrylated PDMS for 2PLP.

**Figure 2:**
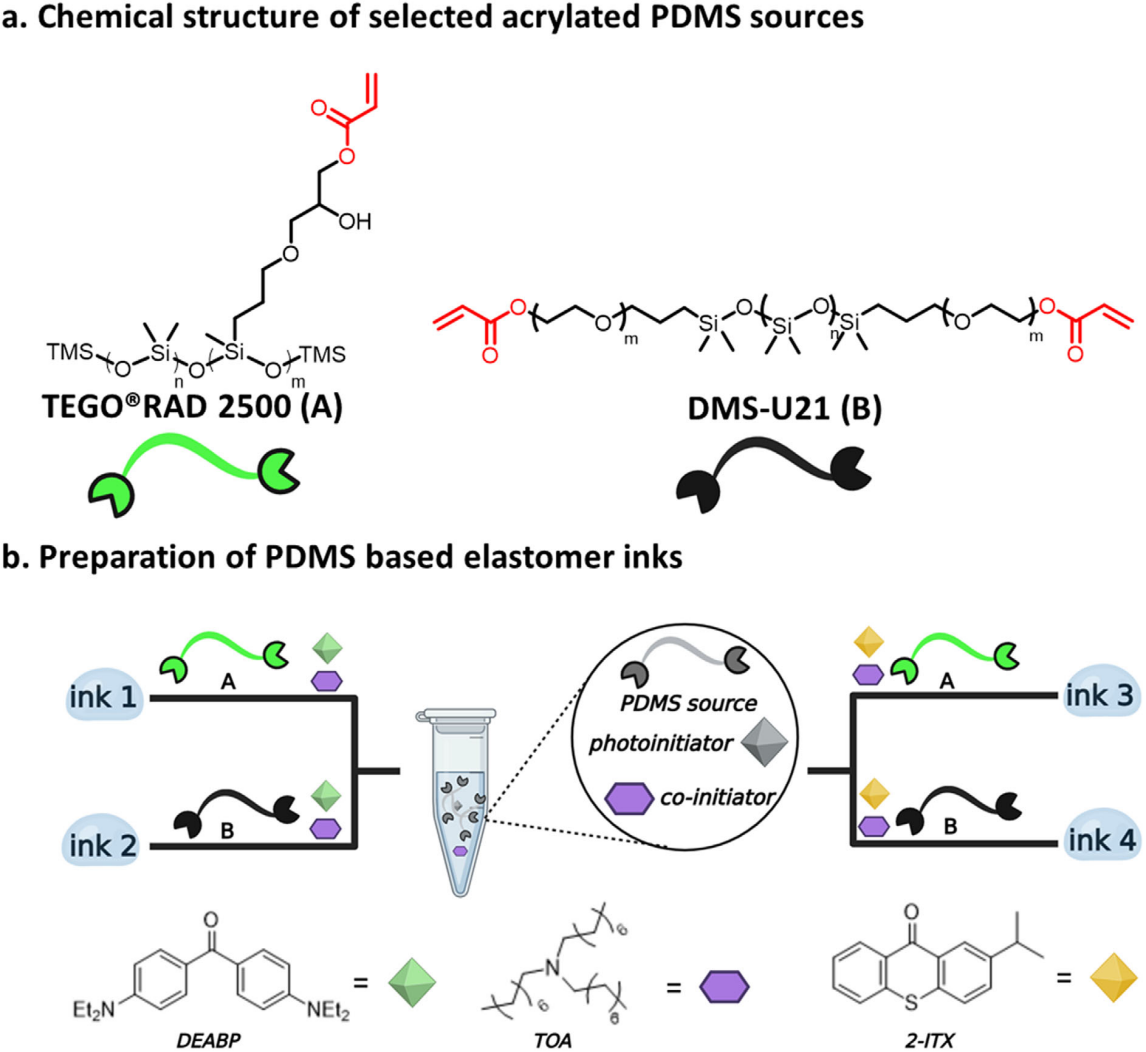
a) Chemical structure of commercially available acrylated PDMS sources selected for this study, TEGORAD 2500 (A) and DMS‐U21 (B). b) Preparation of four 2PLP printable PDMS‐based elastomer inks.

In order to overcome this challenge, ^1^H NMR spectroscopy was used for the chemical characterization of the PDMS sources and in particular, to determine the ratio between the protons from the PDMS main chain (‐Si(CH_3_)_2_) and the acrylate protons (‐CH═CH_2_). A higher degree of acrylation (smaller (‐Si(CH_3_)_2_) to acrylate ratio) is expected to be beneficial for the printability performance due to the ability to form a more crosslinked polymer network. In **Table** **1** (and Figures S1–S5, Supporting Information) the Si(CH_3_)_2_) to (‐CH═CH_2_) ratio for all the PDMS sources was calculated. For this study, the two acrylated PDMS materials that showed the highest degree of acrylation, namely TEGORAD 2500 (**A**) and DMS‐U21 (**B**), selected (**Figure** **2a**) to prepare the series of soft inks (Figure 2b). In addition, a commercially available PDMS based ink IP‐PDMS (Nanoscribe GmbH & Co. KG) was also analyzed by ^1^H NMR as reference and showed a comparable degree of acrylation.

**Table 1:** ^1^H NMR ratio between the Si(CH_3_)_2_) and the CH═CH_2_ protons of commercially available acrylated PDMS sources tested.

Acrylated PDMS source	Si(CH_3_)_2_: CH═CH_2_	Printability
TEGORAD 2250	75:1	No
TEGORAD 2500	22:1	Yes
TEGORAD 2800	144:1	No
DMS‐U21	19:1	Yes
IP‐PDMS (commercial ink)	13:1	Yes

### 2.2 Soft Elastomer Ink Preparation and Microscale 3D Printing

**Figure 3:**
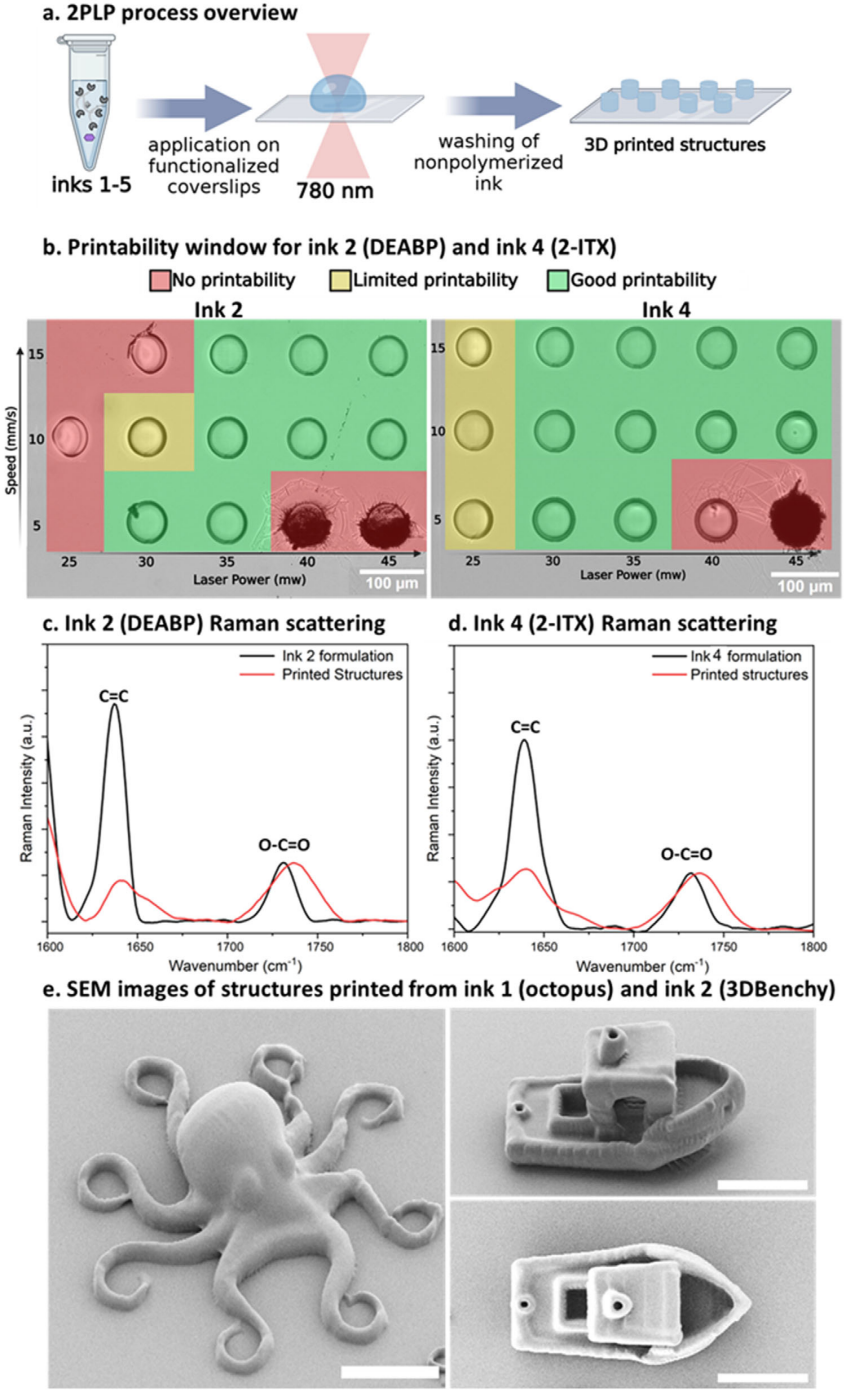
a) 2PLP process overview b) Light microscope images showing the printability window for ink 2 and ink 4 (bottom) using 3D printed pillars (d = 60 µm, h = 15 µm). c) overlayed Raman spectra of ink **2** (**B** + **DEABP**) formulation (black) and printed structures (red). d) overlayed Raman spectra of ink **4** (**B** + **2‐ITX**) formulation (black) and printed structures (red). e) SEM images of 3D printed structures from ink **1** (octopus) and ink **2** (3DBenchy). Scale bars are 20 µm.

The two selected PDMS sources were then formulated into printable inks by blending the selected PDMS source with a photoinitiator and a co‐initiator. It is known that the choice of photoinitiating system can significantly affect the final mechanical properties of the printed structures due to increased conversion of the acrylate functional groups during printing.^[^ ^23^, ^24^ ^]^ Thus, two types of photoinitiators, 2‐isopropylthioxanthone (**2‐ITX**) and 4,4′‐diethylamino‐benzophenone (**DEABP**), with different two‐photon absorption efficiencies (two‐photon cross section values at 800 nm for **2‐ITX** and **DEABP** are 3 and 250 GM, respectively) were selected and mixed with the selected acrylated PDMS materials (**A** and **B**) and **TOA** as co‐initiator (**Figure** **3a**). It is expected that the efficiency of the photoinitiators plays a role in the mechanical properties and inks prepared using the more efficient photoinitiator (**DEABP**, inks **1–2**) will generate stiffer materials compared to inks prepared using the less efficient **2‐ITX** (inks **3–4**).^[^ ^25^ ^]^ Another important criterion for the photoinitiator/material choice is cytocompatibility, since a notable application of 3D printed soft elastomeric microstructures is to mimic the extracellular matrix of cells. Therefore, the two photoinitiators selected are relatively cytocompatible based on literature reports.^[^ ^26^ ^]^

The photoinitiator influence on the materials cross‐linking density was characterized by Raman spectroscopy. By monitoring the decrease of the acrylate C═C signal (1637 cm^−1^) relative to the O─C═O ester signal (1734 cm^−1^) before and after printing between the nonprinted ink formulations and 3D printed pillars (d = 100 µ, h = 20 µm), the degree of acrylate conversion was calculated.^[^ ^20^, ^27^ ^]^ For ink **2**, prepared from PDMS source **B** and the efficient **DEABP** photoinitiator, the relative conversion of the acrylate was calculated to be 83% (Figure 3c; Figure S6, Supporting Information)) while for ink **4**, prepared from the same PDMS source but with **2‐ITX** as photoinitiator, the acrylate conversion was calculated to be 66% (Figure 3d; Figure S7, Supporting Information). The same trend was observed for inks **1** and ink **3,** both prepared from PDMS source **A** but using different photoinitiators. For ink **1**, with **DEABP** the acrylate conversion between the formulation and printed structures was calculated to be 78% (Figure S8, Supporting Information) while for ink **3** with **2‐ITX** the acrylate conversion was found to be 75% (Figure S9, Supporting Information). This Raman characterization shows the increased efficiency of **DEABP** over **2‐ITX**, and suggests that structures printed from ink **1** and ink **2** should yield stiffer structures compared to structures printed using ink **3** and ink **4** due to the increased conversion of the acrylate functional groups during printing. All the inks showed a broad printability window based on qualitative light microscope image analysis between laser powers of 25–45 mW and scanning speeds of 5–15 mm s^−1^ allowing the fabrication of 3D printed pillars (d = 60 µm, h  =  15 µm) via 2PLP (Figure 3a). It is important to note, that the Raman measurements were performed on pillars in the printability region. Outside this region, other factors like photoinitiator and solubility, concentration and photoinitiator to co‐initiator ratio might lead to different degrees of conversion. The performance of the inks was further demonstrated by printing more complex 3D microstructures with fine features or overhanging structures, like the tentacles of the octopus and the chimney on the roof of the 3DBenchy boat depicted in Figure 3e. Overall, the printed structures show good agreement with the original designs (Figures S10,S11, Supporting Information).

### 2.3 Development of an Improved Nanoindentation Protocol for 3D Printed Elastomers at the Microscale

**Figure 4:**
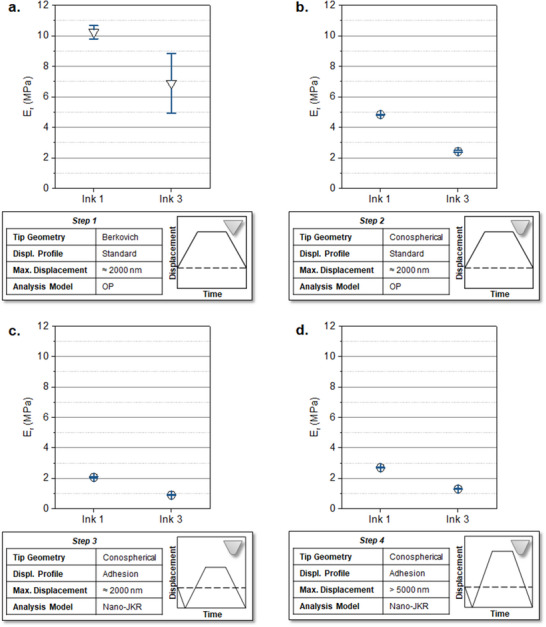
Nanoindentation results of the stepwise developed nanoindentation protocol for tacky 3D printed elastomers at the microscale. a) Step 1: *“Standard”* displacement‐controlled trapezoidal displacement profile using a Berkovich tip and the OP model for analysis. b) Step 2: *“Standard”* displacement‐controlled trapezoidal displacement profile using a conospherical tip (tip radius =  10.26 µm) and the OP model for analysis. c) Step 3: Adhesion‐adapted displaced controlled displacement profile with lift‐off segments at the start and end of the indent and the nano‐JKR model for analysis. d) Step 4: Deeper adhesion‐adapted indents to find the optimal indentation depth.

Using the developed inks, a nanoindentation protocol was stepwise designed and extensively tested in order to bridge the gap in the mechanical properties characterization of soft 3D printed materials at the microscale. Usually, reported nanoindentation protocols in literature are based on standard methods that employ a Berkovic tip and a trapezoidal displacement profile to indent the microscale 3D printed materials.^[^ ^6^, ^23^, ^28^ ^]^ Thus, using the optimal printing parameters for ink** 1** and ink **3** as exemplary materials, “standard” nanoindentation experiments employing a Berkovich tip were performed on sets of 3D printed cylindrical pillars (d = 100 µm, h = 20 µm) as the first step in our approach (**Figure** **4a**; Figures S12 and S13a, Table S1, Supporting Information). The pillars were indented to a peak displacement of 2000 nm. For analysis, the OP model was employed.^[^ ^22^ ^]^ No signs of visual damage were observed after indenting the samples (Figure S14, Supporting Information). The reduced moduli obtained for the pillars printed with ink** 1** and ink** 3** were 10.23 ± 0.44 and 6.88 ± 1.95 MPa, respectively. Notably, the reduced modulus for the structures printed with ink** 3** exhibited a high standard deviation, accounting for nearly 30% of the average value. The pronounced variability in the results suggested that the use of a such “standard” nanoindentation procedure is suboptimal for soft materials. Unlike rigid microscale materials, that show little to no adhesion,^[^ ^12^, ^13^ ^]^ elastomeric‐based microstructures are significantly affected by adhesive forces between their tacky surface and the (sharp) indenter tip. These interactions can lead to distortions and overestimation of the elastic modulus. Thus, there is a clear necessity to establish a more reliable protocol.

To begin with the optimization of our new protocol, the same “standard” indentation procedure of step 1 was repeated in step 2 using a conospherical tip with a 10.26 µm radius (Figure 4b; Figures S12,S13b, Supporting Information). Using this approach, the obtained reduced moduli were 4.83 ± 0.03 and 2.42 ± 0.06 MPa for structures printed with ink** 1** and ink** 3,** respectively. Next, to account for adhesion forces, an adapted trapezoidal displacement profile was introduced in step 3. This nanoindentation profile incorporates an initial lift‐off segment of 4000 nm, followed by a loading segment, a hold period at a peak displacement of ≈2000 nm and an unloading segment that includes an additional 4000 nm lift‐off from the sample (Figure 4c; Figure S13c, Supporting Information). One key advantage of employing conospherical tips is the ability to use analytical methods such as the nano‐JKR model, which is widely applied for analyzing nanoindentation load–displacement curves in macroscopic PDMS samples.^[^ ^14^, ^18^, ^29^, ^30^ ^]^ This method, originally reported by Kohn and Ebenstein,^[^ ^31^ ^]^ mitigates adhesion effects between the nanoindenter tip and the sample, which can otherwise lead to significant overestimation of the modulus when analyzed using traditional models like OP, as seen on the first step. Again, no signs of visual damage were observed after indenting the samples (Figure S14, Supporting Information). Using this approach, the reduced modulus determined was 2.07 ± 0.05 MPa for ink** 1** and 0.90 ± 0.04 MPa for ink** 3**. Comparing to step 1, these results reveal a significant discrepancy: modulus values obtained with the Berkovich tip and OP model were nearly five times higher for ink 1 (10.23 ± 0.44 MPa vs 2.07 ± 0.05 MPa, 80% reduction from step 1) and almost eight times higher ink** 3** (6.88 ± 1.95 MPa vs 0.90 ± 0.04 MPa, 85% reduction from step 1). Similar observations have been reported by Gupta et al. when comparing the modulus of macroscopic PDMS samples analyzed with the Hertz model versus when calculated with the JKR model.^[^ ^32^ ^]^ These findings emphasize the critical importance of using a conospherical tip instead of a Berkovich tip and accounting for adhesion effects at the tip‐sample interface to ensure reliable modulus determination of microscale 3D printed elastomers.

Last, to determine the optimal peak displacement and investigate possible substrate effects^[^ ^14^, ^33^ ^]^—given that the soft elastomeric pillars are printed onto a glass microscope slide—the adapted displacement profile of step 3 was performed for peak displacements exceeding 5000 nm, corresponding to more than 25 % of the total pillar height (Figure 4d; Figure S13d, Supporting Information). Under these conditions, the reduced modulus of the structures printed with ink** 1** was 2.70 ± 0.02 MPa, representing a 30.7% increase compared to measurements performed peak displacements of ≈2000 nm. Similarly, for ink **3**, the modulus increased by 44.2%, reaching 1.30 ± 0.03 MPa. Although the substrate effect is less pronounced than the influence of the indenter tip geometry and nanoindentation protocol, it still contributes to modulus overestimation, particularly for materials with lower stiffness. The data indicate that the softer the material, the greater is the impact of substrate effects on deep nanoindentation measurements. To minimize these artifacts, the optimal peak displacement should remain below 10% of the total pillar height as suggested by previous studies,^[^ ^14^, ^33^ ^]^ which in this study corresponds to a maximum of 2000 nm.

In summary, the optimal protocol for measuring the elastic modulus of tacky, 3D printed soft elastomers at the microscale includes:

1) Performing the nanoindentation experiments with displacement control feedback to ensure a constant strain rate (max. displacement/segment time) is applied to all samples, measure properties at the same peak displacement regardless of material stiffness, and reveal adhesion forces at the tip‐sample interface.
2) Using a conospherical probe tip (r  =  10.26 µm) instead of Berkovich tip.
3) Employing and adapted displacement profile with lift‐off segments at both the start and the end of the indent.
4) Setting the peak displacement to ≤ 10% of the total sample thickness (i.e., in this case, ≈2000 nm) to mitigate substrate effects.
5) Applying the nano‐JKR model, which effectively accounts for adhesive forces between the tip and the pillar surface.

**Figure 5:**
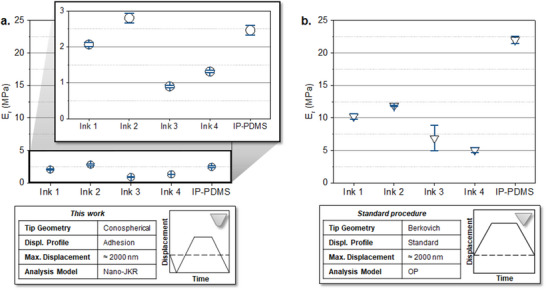
Comparison of the nanoindentation results of a) the optimized protocol employing a conospherical tip (radius = 10.26 µm) with a displacement profile including lift‐off segments at the start and end of the indent and the nano‐JKR model for analysis, and b) the “standard” protocol employing a Berkovich tip with a trapezoidal displacement profile and the OP model for analysis.

The optimized protocol was then tested on round pillars of identical dimensions but 3D printed with the rest of the developed inks (inks **1‐4**, **Figure** **5a**; Table S2, Supporting Information). For comparison, the same structures were tested using the “standard” nanoindentation protocol (Figure 5b).^[^ ^11^, ^20^, ^21^, ^22^ ^]^ For the inks prepared using **DEABP** as photoinitiator (inks** 1‐2**) the reduced moduli determined using the optimized protocol were 2.07 ± 0.05 and 2.80 ± 0.14 MPa, respectively. In contrast, the values obtained using the “standard” protocol were significantly higher: 10.23 ± 0.44 and 11.87 ± 0.09 MPa (Figure 5b). This fivefold overestimation highlights the influence of adhesion forces between the indenter tip and the surface of the elastomers on the measured values, which if unaccounted for, lead to a significant overestimation of the mechanical properties. For the inks prepared using **2‐ITX** as photoinitiator (inks** 3‐4**), the optimized protocol yielded moduli of 0.90 ± 0.04 and 1.31 ± 0.03 MPa, respectively. The variation of the PDMS source between **A** and **B**, (22:1 and 19:1 ^1^H‐NMR Si (**CH_3_**)_2_ to **CH**═**CH_2_** ratio, respectively) had a less pronounced effect on the modulus compared to the photoinitiator selection. The discrepancies between optimized and standard protocols remained substantial, showing modulus overestimations of approximately eightfold for ink** 3** (6.88 ± 1.95 MPa) and approximately fivefold for ink** 4** (5.06 ± 0.35 MPa).

To further verify the method, the optimized protocol was applied to measure the modulus of a commercial reference, IP‐PDMS (Nanoscribe GmbH & Co. KG), a 2PLP PDMS based printing material known to be adhesive.^[^ ^16^ ^]^ The optimized protocol yielded a reduced modulus of 2.46 ± 0.13 MPa, whereas when employing the “standard” procedure, the modulus was estimated to be 21.99 ± 0.59 MPa—an order of magnitude difference. The reported literature stiffness values for IP‐PDMS structures under comparable printing conditions range from 5–11 MPa.^[^ ^16^ ^]^ And although these values, obtained using different geometries, they are still 2–5 fold larger than measured using a protocol that is suitable to soft and tacky 3D printed microscale materials. The observed discrepancies between the reported values for IP‐PDMS and the obtained values can be attributed to the use of traditional nanoindentation methods, such as the OP and the Hertz methods,^[^ ^16^ ^]^ which assume neglectable adhesion forces. Adhesion induces pulling forces during sample approach and withdrawal, causing an overestimation of the modulus when ignored (Figure S15, Supporting Information).^[^ ^29^, ^30^, ^31^, ^32^ ^]^ Numerical simulations that explicitly account for adhesive tip‐sample interactions confirmed their significant effect of adhesion on modulus estimation for macroscopic PDMS samples.^[^ ^15^ ^]^ These results further underscore the crucial role of adhesion forces in the mechanical characterization of 3D printed microscale elastomers and highlight the necessity of the developed nanoindentation protocol.

## 3 Conclusion

This work presents the standardization and benchmarking of a nanoindentation protocol for 3D printed elastomeric microstructures, addressing key challenges associated with adhesion forces at the tip‐sample interface and substrate effects. The optimized nanoindentation method employs a conospherical indenter tip (r  =  10.26 µm), a modified trapezoidal displacement profile lift‐off segments at the start and end of the indentation, and the utilization of the nano‐JKR model for data analysis, allowing for consistent elastic modulus determination of the 3D printed microstructures. Via thorough testing with four newly developed and readily accessible PDMS‐based elastomer inks for 2PLP, the protocol effectively measured modulus ranging from 0.9 to 3 MPa. Comparative analysis using a “standard” nanoindentation protocol—which uses a Berkovich indenter tip, an unmodified trapezoidal load function and OP model for data analysis—revealed that traditional methods lead to a significant overestimation of the elastic modulus by up to an order of magnitude because they do not take adhesion into account. The known influence of adhesion forces on nanoindentation measurements on 3D printed soft materials has been overlooked. One reason is that adhesion appears as a negative load during unloading in a load–displacement curve, which is missed when performing nanoindentation in load‐controlled experiments. Furthermore, conventionally used models, like the Hertzian contact theory or the OP method assume negligible adhesion, leading to a systematic overestimation of the elastic modulus in such cases. These findings highlight the importance of carefully selecting a nanoindentation protocol when studying the mechanical properties of complex materials, such as 3D microprinted elastomers. Differences in sample size, experimental parameters, indenter tip geometry, and analysis models can significantly affect the modulus determination, underlining the urgent need for standardized methodologies in the characterization of soft 3D printed materials at the microscale, to ensure comparability and accuracy across studies in this field. Future efforts will focus on extending the presented protocol to additional soft material systems and environmental conditions. This will include hydrogels for use bioscience applications.

## 4 Experimental Section

### Materials

All materials were used as received without further purification unless otherwise is stated. Trioctylamine (98%, Sigma–Aldrich) 3‐(trimethoxysilyl) propyl methacrylate (98%, Sigma–Aldrich); chloroform‐d (99.8 atom % D, Sigma–Aldrich); Acrylated PDMS resins (3‐Acryloxi‐2‐Hydroxypropoxypropyl) terminated polydimethylsiloxane, 60–140 cSt (DMS‐U21) was purchased from Gelest and used without further manipulation. TEGORAD2500 were supplied by Evonik and used without further manipulation. IP‐PDMS was purchased from Nanoscribe GmbH & Co. KG (Karlsruhe, Germany) and was employed as a commercial reference.

### Methods

*Commercial PDMS screening*: Commercially available acrylated PDMS products were screened by mixing 200 mg of the corresponding acrylated PDMS, 49.3 µL of trioctylamine and 4.0 mg of 2‐isopropylthioxanthone. The mixture was sonicated at 50 °C for 30 min or until homogenous mixture was obtained. The formulations were then applied on functionalized coverslips and printing attempts with different printing parameters were performed using the specified 2PLP procedure. Formulations from commercially available PDMS sources that showed potential printability were further optimized.

*Ink preparation*: Inks were prepared by weighing suitable amount of photoinitiator (0.4% w/w) for 4,4′‐Bis(diethylamino)benzophenone and 1.2% w/w for 2‐isopropylthioxanthone) into a 2 mL vial. Then trioctylamine was added, followed by the cross‐linkable PDMS. The inks were sonicated in an ultrasonic bath at 50 °C for 1 h until the ink became homogenous and printed directly after preparation.

**Ink 1**: 1.0 mg 4,4′‐Bis(diethylamino)benzophenone (0.4% w/w), 49.3 µL trioctylamine (16.6% w/w), 200 mg TEGO RAD 2500 (83.0% w/w).

**Ink 2**: 1.0 mg 4,4′‐Bis(diethylamino)benzophenone (0.4% w/w), 49.3 µL trioctylamine (16.6% w/w), 200 (3‐Acryloxi‐2‐Hydroxypropoxypropyl) terminated polydimethylsiloxane, 60–140 cSt (DMS‐U21, 83.0% w/w).

**Ink 3**: 3.0 mg 2‐isopropylthioxanthone (1.2% w/w), 49.3 µL trioctylamine (16.4% w/w), 200 mg TEGO RAD 2500 (82.4% w/w).

**Ink 4**: 3.0 mg 2‐isopropylthioxanthone (1.2% w/w), 49.3 µL trioctylamine (16.4% w/w), 200 mg (3‐Acryloxi‐2‐Hydroxypropoxypropyl) terminated polydimethylsiloxane, 60–140 cSt (DMS‐U21, 82.4% w/w).

*^1^H NMR*: Measurements of the samples in deuterated chloroform (CDCl_3_) were performed using a Bruker Avance III 300 MHz equipped using 5 mm BBO BB probe at 25 °C.

*Silanization*: Glass coverslips (22 × 22 mm, 170 µm thickness) were thoroughly cleaned using isopropanol and acetone and dried under a stream of air. Then, the surface was activated for 1 min using a piezobrush PZ2 handheld plasma cleaner (Relyon Plasma GmbH, TDK Group Company). The coverslips were then immersed in a 4 mm solution of 3‐(trimethoxysilyl)propyl acrylate in toluene overnight. Finally, the functionalized coverslips were washed with toluene (x2), acetone and dried under a stream of air.

*PDMS molds* were prepared according to the instructions on the data sheet.^[^ ^34^ ^]^ The liquid prepolymer base and the cross‐linking curing agent were thoroughly mixed in a 10:1 ratio. After mixing, the PDMS was degassed under vacuum until no more air bubbles appeared. Then, 7 g of the mixture was cast into round Petri dishes (92×16 mm^2^) and cured under ambient conditions for at least 72 h. Once cured, the rectangular molds were cut from the PDMS sheet with a scalpel (10×10 mm^2^ outside dimensions and a 5×5 mm^2^ inner cavity).

*Two‐Photon 3D Laser printing*: 2PLP was performed on the commercially available setup Photonic Professional GT2 (Nanoscribe GmbH & Co. KG) in oil immersion configuration with a femtosecond laser (𝜆 = 780 nm) focused through a 25× oil objective (NA = 0.8; WD =  380 µm; Zeiss). The instrument has a maximum output of 50 mW. The printing GWL files for 3D structure fabrication were generated from STL files of the desired geometries with the help of the Describe software (Nanoscribe GmbH & Co. KG). Slicing and hatching were set both to 0.5 µm. Printing was performed with laser powers in the range of 30 – 45 mW and scanning speeds between 1000 – 20 000 µm s^−1^. Silanized coverslips were attached by tape onto a commercial sample holder (Nanoscribe GmbH & Co. KG) for oil immersion mode. Immersion oil was added on the unfunctionalized slide surface, and the ink on the functionalized one to ensure good adhesion of the 3D printed microstructures. To maintain the environmental conditions of the ink as reproducible as possible, the ink was loaded into a PDMS mold and covered with a 10 mm diameter circular coverslip during printing. After printing, the circular coverslip and PDMS mold were removed, and the excess oil was removed using isopropanol. If not stated otherwise, the printed samples were developed in isopropanol for 10 min, and then the samples were submerged again for 2 min in fresh isopropanol for rinsing.

*Light Microscopy*: The 3D printed microstructures were imaged on an Axio Imager M2 microscope (Carl Zeiss Microscopy) equipped with an LD Plan‐Neofluar 20×/0.4 KorrPh M27 objective and an Axiocam 705 microscope camera.

*Scanning Electron Microscopy*: SEM was performed using a field‐emission scanning electron microscope (Ultra 55, Carl Zeiss Microscopy) at a primary electron energy of 3 keV. Prior to imaging, the 3D printed microstructures were sputter coated with a 12 nm layer of Pt/Pd (80:20).

*Nanoindentation*: Nanoindentation was performed on 100 (d) x 20 (h) µm 3D printed cylindrical pillars using a Bruker Hysitron TI 980 Nanoindenter equipped with a BioXR transducer. Two different tip geometries were employed for this study as stated in the Results Section (2.3. Development an improved nanoindentation protocol for 3D printed elastomers at the microscale): a standard Berkovich tip and a 10.26 µm radius conospherical tip, both made of diamond. Prior to the measurements, the indentation tip was calibrated against air and the tip area function was calculated. All indents were performed in displacement control. The data were then analyzed using two methods depending on the application: the Oliver–Pharr (OP) method^[^ ^22^ ^]^ or the nano‐Johnson–Kendall–Roberts (nano‐JKR) model.^[^ ^14^, ^18^, ^29^, ^30^ ^]^ Two displacement profiles were performed. Standard trapezoidal displacement profiles with a peak displacement of 2000 nm and adapted profile, that included lift‐off segments to account for adhesion forces: After the surface was first detected, the tip was lifted off from the surface to a height of 4000 nm, well outside of the adhesive interaction zone, and finally approached again, to capture the full interaction between the nanoindenter tip and the sample. The samples were then indented to a peak displacement of ≈2000 nm. After indentation, the tip was withdrawn from the sample to the same lift‐off height. The data were then analyzed using the Origin App “Soft Matter Analysis” that belongs to the Bruker's Tribo iQ suite of the technique‐specific software applications. All measurements were performed in triplicates, and the results are shown as mean ± SD.

*Confocal Raman Spectroscopy*: Pillars (d = 100 µm, h = 20 µm) were fabricated. Raman spectra were collected with a confocal Raman spectrometer (Renishaw InVia Reflex) in backscattering configuration equipped with a 532 nm laser and a 50× long working distance objective (Olympus, NA = 0.5). A calibration with a silicon wafer at 520.6 cm^−1^ was performed prior to each measurement. Each spectrum was recorded with 75 s of bleaching time, 2 s of integration time, 20 accumulations, and laser power of 100%. For all samples, n = 3 measurements were performed at random points, with 3 repeats per scan speed and laser power parameter, with a focus point centered 1 µm below the surface to exclude an influence of surface inhomogeneities. The mean for the three spectra for each printed structure was calculated, and the average of the three measurements was used for standard deviation calculations. Before averaging, each spectrum was baseline corrected and smoothened (Savitzky–Golay). Conversion calculations were performed based on previously published work.^[^ ^20^ ^]^

## Conflict of Interest

The authors declare no conflict of interest.

## Supporting information



Supporting Information

## Data Availability

The data that support the findings of this study are openly available in heiDATA, the Open Research Data institutional repository for Heidelberg University at https://doi.org/10.11588/DATA/4OZZTW.

## References

[1] R. J. Spontak , N. P. Patel , Curr. Opin. Colloid Interface Sci. 2000, 5, 333.

[2] a) S. C. Ligon , R. Liska , J. Stampfl , M. Gurr , R. Mülhaupt , Chem. Rev. 2017, 117, 10212; 28756658 10.1021/acs.chemrev.7b00074 PMC5553103

[3] a) J. Herzberger , J. M. Sirrine , C. B. Williams , T. E. Long , Prog. Polym. Sci. 2019, 97, 101144;

[4] K. Navindaran , J. S. Kang , K. Moon , J. Mech. Behav. Biomed. Mater. 2023, 138, 105575. 36470112 10.1016/j.jmbbm.2022.105575

[5] a) J. Bauer , A. Guell Izard , Y. Zhang , T. Baldacchini , L. Valdevit , Adv. Mater. Technol. 2019, 4, 1900146;

[6] T. Koch , W. Zhang , T. T. Tran , Y. Wang , A. Mikitisin , J. Puchhammer , J. R. Greer , A. Ovsianikov , F. Chalupa‐Gantner , M. Lunzer , Adv. Mater. 2024, 36, 2308497. 10.1002/adma.202308497 38303404

[7] N. Rohbeck , R. Ramachandramoorthy , D. Casari , P. Schürch , T. E. Edwards , L. Schilinsky , L. Philippe , J. Schwiedrzik , J. Michler , Mater. Des. 2020, 195, 108977.

[8] a) C.‐S. Shin , T.‐J. Li , C.‐L. Lin , Micromachines 2018, 9, 615;30467303

[9] D. M. Ebenstein , L. A. Pruitt , Nano Today 2006, 1, 26.

[10] a) A. C. Fischer‐Cripps , Introduction to Contact Mechanics, Springer US; Imprint: Springer, New York, NY 2007;

[11] C. E. Hoffler , X. E. Guo , P. K. Zysset , S. A. Goldstein , J. Biomech. Eng. 2005, 127, 1046. 16502646 10.1115/1.2073671

[12] L. Angker , M. V. Swain , J. Mater. Res. 2006, 21, 1893.

[13] G. Lewis , J. S. Nyman , J. Biomed. Mater. Res., Part B 2008, 87, 286. 10.1002/jbm.b.31092 18395829

[14] S. E. Arevalo , D. M. Ebenstein , L. A. Pruitt , J. Mech. Behav. Biomed. Mater. 2022, 134, 105384. 35961240 10.1016/j.jmbbm.2022.105384

[15] a) A. Sharfeddin , A. A. Volinsky , G. Mohan , N. D. Gallant , J. Appl. Polym. Sci. 2015, 132, 42680;

[16] P. F. J. van Altena , A. Accardo , Polymers 2023, 15, 1816. 37111964 10.3390/polym15081816 PMC10144803

[17] a) M. Hippler , E. D. Lemma , S. Bertels , E. Blasco , C. Barner‐Kowollik , M. Wegener , M. Bastmeyer , Adv. Mater. 2019, 31, 1808110; 10.1002/adma.201808110 30793374

[18] D. M. Ebenstein , J. Mater. Res. 2011, 26, 1026.

[19] K. L. Johnson , K. Kendall , A. D. Roberts , Proc. R. Soc. London, Ser. A 1971, 324, 301.

[20] S. O. Catt , M. Hackner , J. P. Spatz , E. Blasco , Small 2023, 19, 2300844. 10.1002/smll.202300844 37078908

[21] C. A. Spiegel , M. Hackner , V. P. Bothe , J. P. Spatz , E. Blasco , Adv. Funct. Mater. 2022, 32, 2110580.

[22] W. C. Oliver , G. M. Pharr , J. Mater. Res. 1992, 7, 1564.

[23] Q. Hu , G. A. Rance , G. F. Trindade , D. Pervan , L. Jiang , A. Foerster , L. Turyanska , C. Tuck , D. J. Irvine , R. Hague , et al., Addit. Manuf. 2022, 51, 102575.

[24] D. T. Meiers , M. Rothammer , M. Maier , C. Zollfrank , G. von Freymann , Adv. Eng. Mater. 2023, 25, 2201688.

[25] a) P. Kiefer , V. Hahn , M. Nardi , L. Yang , E. Blasco , C. Barner‐Kowollik , M. Wegener , Adv. Optical Mater. 2020, 8, 2000895;

[26] B. Zeng , Z. Cai , J. Lalevée , Q. Yang , H. Lai , P. Xiao , J. Liu , F. Xing , Toxicol. In Vitro 2021, 72, 105103. 33516932 10.1016/j.tiv.2021.105103

[27] a) M. Diamantopoulou , N. Karathanasopoulos , D. Mohr , Addit. Manuf. 2021, 47, 102266;

[28] R. Srinivasaraghavan Govindarajan , S. Sikulskyi , Z. Ren , T. Stark , D. Kim , Polymers 2023, 15, 4377. 38006101 10.3390/polym15224377 PMC10675433

[29] Y. Cao , D. Yang , W. Soboyejoy , J. Mater. Res. 2005, 20, 2004.

[30] F. Carrillo , S. Gupta , M. Balooch , S. J. Marshall , G. W. Marshall , L. Pruitt , C. M. Puttlitz , J. Mater. Res. 2005, 20, 2820.

[31] J. C. Kohn , D. M. Ebenstein , J. Mech. Behav. Biomed. Mater. 2013, 20, 316. 23517775 10.1016/j.jmbbm.2013.02.002

[32] S. Gupta , F. Carrillo , C. Li , L. Pruitt , C. Puttlitz , Mater. Lett. 2007, 61, 448.

[33] a) A. C. Chang , J. D. Liao , B. H. Liu , Mech. Mater. 2016, 98, 11;

[34] The Dow Chemical Company, Technical Data Sheet Sylgard 184, Form No. 11‐3184‐01 C 2018, https://www.dow.com/en‐us/document‐viewer.html?randomVar=945999166439461542&docPath=/content/dam/dcc/documents/11/11‐3184‐01‐sylgard‐184‐elastomer.pdf.

