# Supplementary material for: Benchmarking Mechanical Properties of 3D Printed Elastomeric Microstructures
Source: Small Methods. 2025 Jun 12;10(2):2500432. doi: 10.1002/smtd.202500432 (PMC12825332; doi:10.1002/smtd.202500432)
Supplement: Supplementary file 1 — Supporting Information [file SMTD-10-2500432-s001.docx]

Supporting Information

Benchmarking Mechanical Properties of 3D Printed Elastomeric Microstructures

Or Eivgi^†^, Clara Vazquez-Martel^†^, Jaroslav Lukeš and Eva Blasco*

Or Eivgi, Clara Vazquez-Martel and Eva Blasco
Institute for Molecular Systems Engineering and Advanced Materials (IMSEAM)
Heidelberg University
Im Neuenheimer Feld 225
69120 Heidelberg, Germany

Jaroslav Lukeš
Bruker Nano Surfaces and Metrology
Technická 4, Prague 6
160 00, Czech Republic

^†^These authors contributed equally.

*Corresponding author: eva.blasco@oci.uni-heidelberg.de

This document contains **13** pages (including this cover sheet), **15** Figures and **2** Tables.

**TABLE OF SUPPLEMENTARY CONTENT**

| 1. ***^1^H NMR spectra of commercially available acrylated PDMS sources***   **Figure S1 –** ^1^H NMR Spectrum of commercially available acrylated PDMS source TEGO^®^RAD 2250  **Figure S2 –** ^1^H NMR Spectrum of commercially available acrylated PDMS source TEGO^®^RAD 2500  **Figure S3 –** ^1^H NMR Spectrum of commercially available acrylated PDMS source TEGO^®^RAD 2800  **Figure S4 –** ^1^H NMR Spectrum of commercially available acrylated PDMS source DMS-U21  **Figure S5 –** ^1^H NMR Spectrum of commercially available 2PLP ink IP-PDMS | ***Page S2-S6*** |
| --- | --- |
| 1. ***Raman spectra and calculation of acrylate conversion***   **Figure S6** **–** Raman spectra of ink 2 formulation (left) and ink 2 printed structures (right) and calculation of acrylate conversion.  **Figure S7** **–** Raman spectra of ink4 formulation (left) and ink 4 printed structures (right) and calculation of acrylate conversion.  **Figure S8** **–** Raman spectra of ink 1 formulation (left) and ink 1 printed structures (right) and calculation of acrylate conversion.  **Figure S9** **–** Raman spectra of ink 3 formulation (left) and ink 3 printed structures (right) and calculation of acrylate conversion. | ***Page S7-S8*** |
| 1. ***Printability evaluation***   **Figure S10 –** Printability evaluation of octopus printed using ink 1. Top: SEM Images and measurement marks for xy and z axis. Bottom: STL model file with designed dimensions.  **Figure S11 –** Printability evaluation of 3DBenchy printed using ink 2. Top: SEM Images and measurement marks for xy and z axis. Bottom: STL model file with designed dimensions. | ***Page S9*** |
| 1. ***Development an improved nanoindentation protocol for 3D printed elastomers at the microscale***   **Figure S12 –** Detailed specifications and microscope images of the probe tips used in this study: (a) Berkovich and (b) conospherical tip.  **Figure S13 –** Exemplary load-displacement response of ink 1 during the stepwise optimization of the nanoindentation protocol.  **Table S1 –** Reduced moduli E_r_ measured via nanoindentation while performing the stepwise optimization of the nanoindentation protocol for tacky 3D printed elastomers at the microscale (ink 1 and ink 4).  **Figure S14 –** Comparison of three 3D printed pillars printed using ink 1, before and post nanoindentation  **Figure S15 –** Comparison of the nanoindentation results of the ‘standard’ protocol employing a Berkovich tip with a trapezoidal displacement profile (DP) and the Oliver-Pharr (OP) model, the ‘standard’ trapezoidal DP using a conospherical tip (tip radius R = 10.26 µm) and the Hertzian model for analysis, and the adhesion-adapted DP and the nano-Johnson-Kendall-Roberts (nano-JKR) model.  **Table S2** **–** Reduced moduli E_r_ measured via the optimized protocol (this work) compared to the moduli obtained via the ‘standard’ protocol. | ***Page S10-S12*** |

1. **^1^H NMR spectra of commercially available PDMS sources**

**
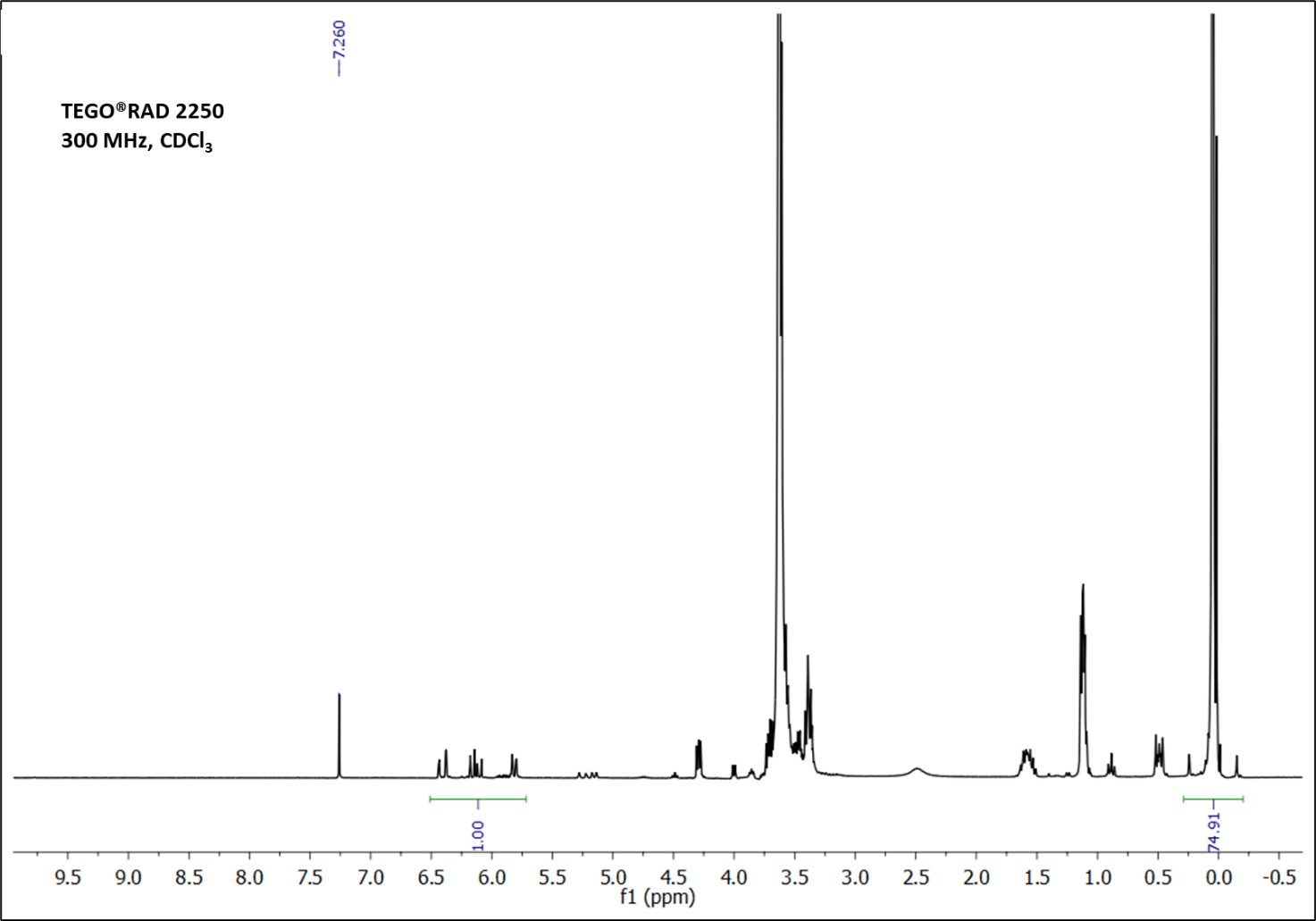
**

***Figure S1.*** *^1^H NMR spectrum of commercially available acrylated PDMS source TEGO^®^RAD 2250.*


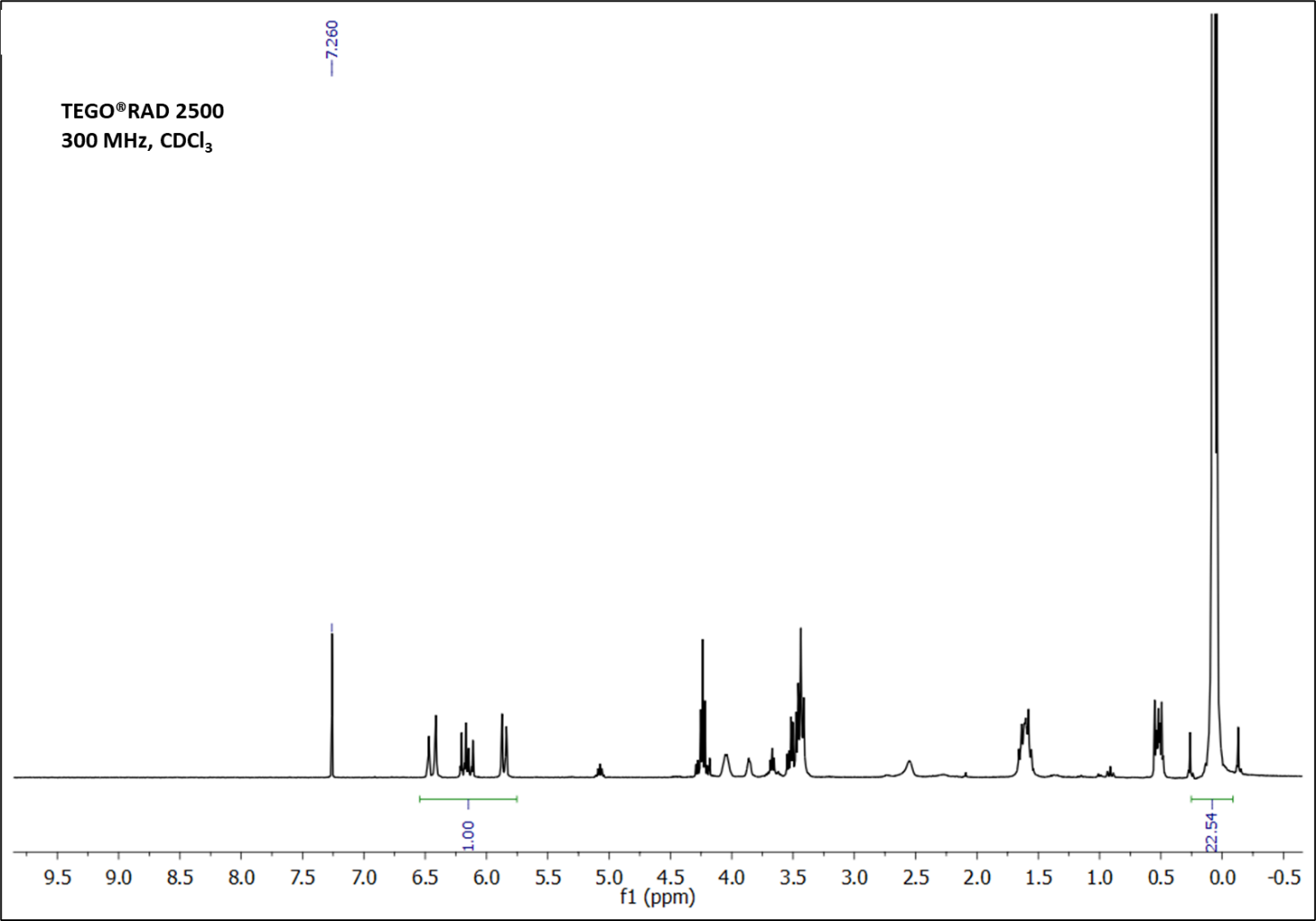


***Figure S2.*** *^1^H NMR spectrum of commercially available acrylated PDMS source TEGO^®^RAD 2500.*


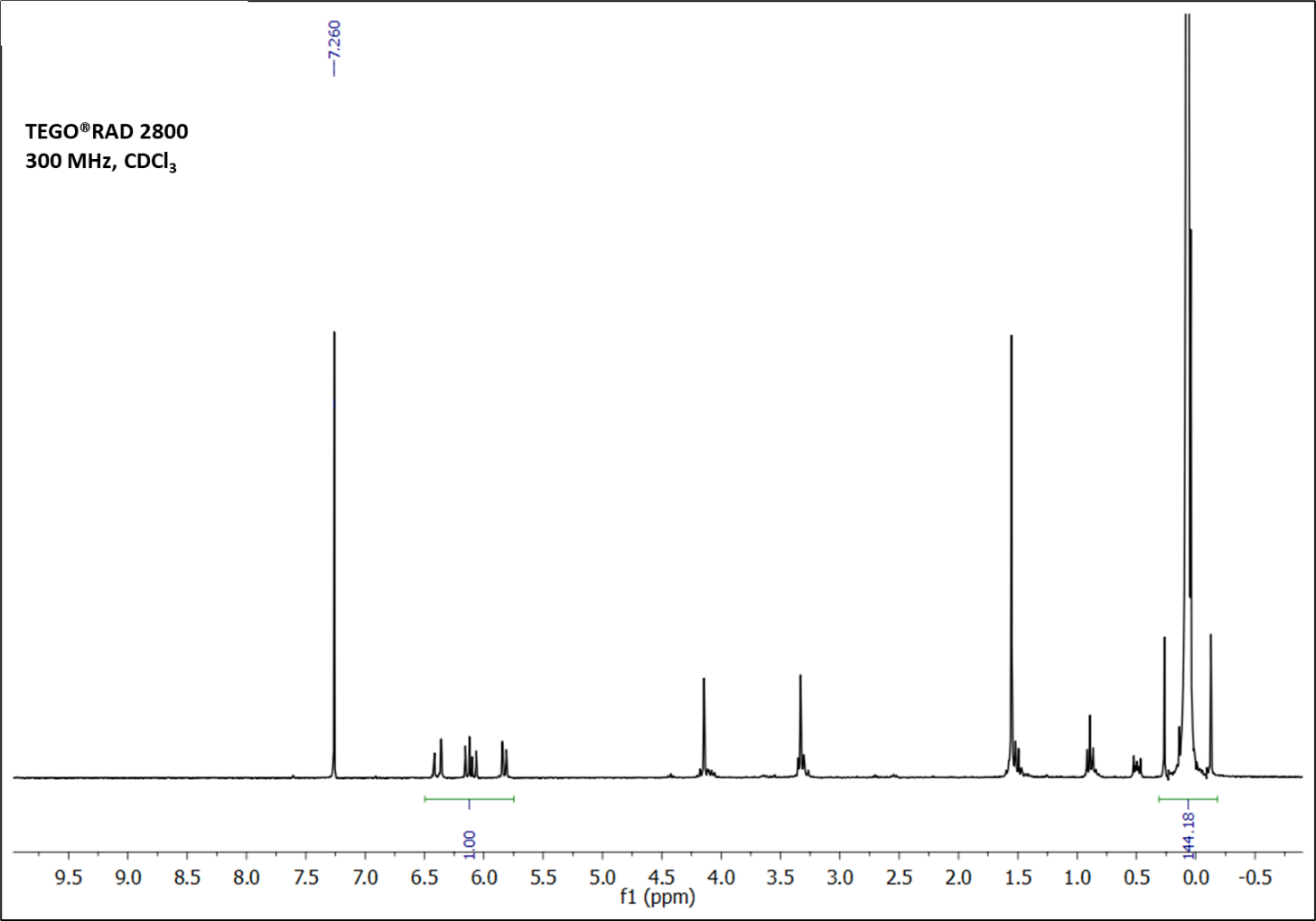


***Figure S3.*** *^1^H NMR spectrum of commercially available acrylated PDMS source TEGO^®^RAD 2800.*


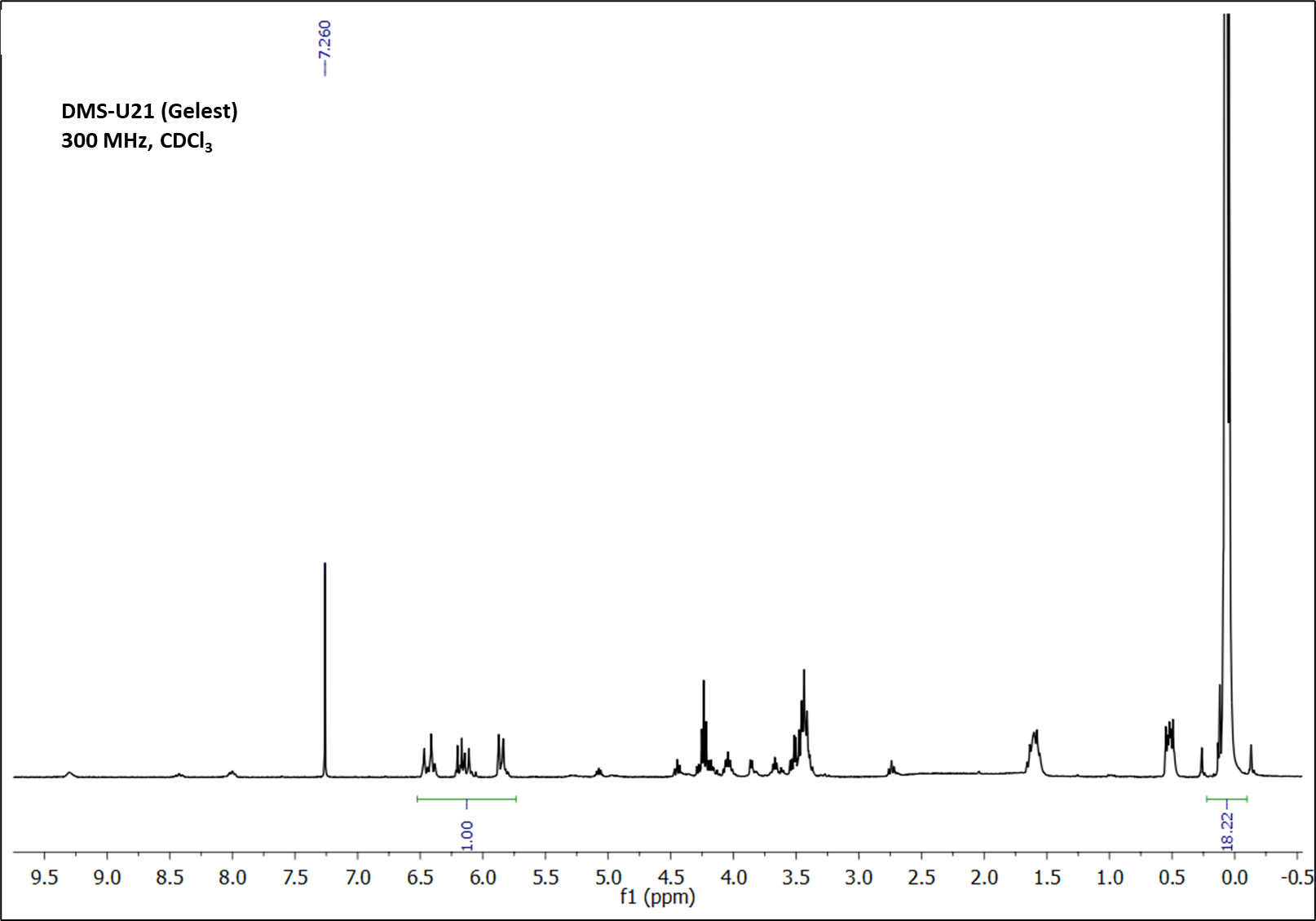


***Figure S4.*** *^1^H NMR spectrum of commercially available acrylated PDMS source DMS-U21 (Gelest).*

*
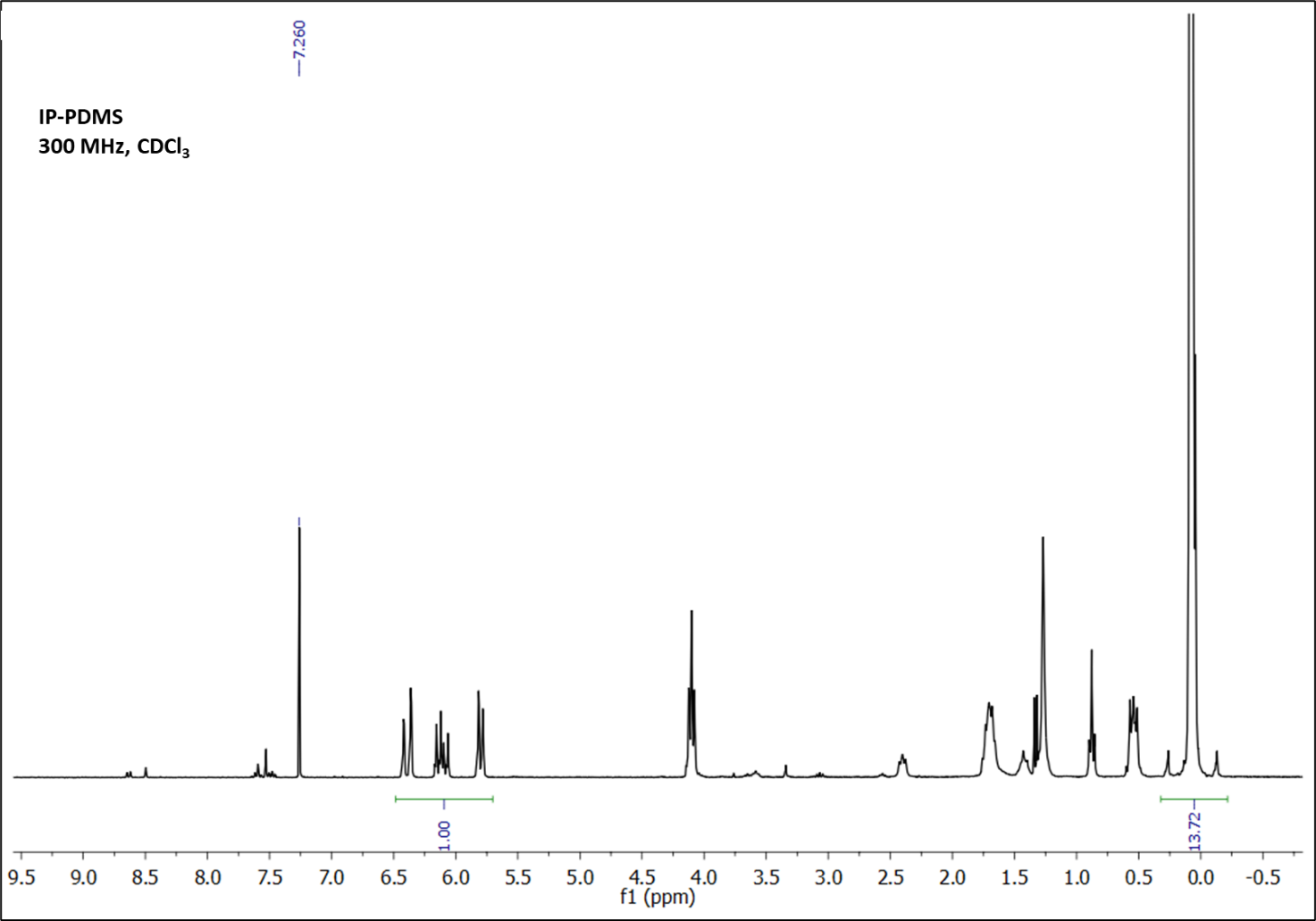
*

***Figure S5.*** *^1^H NMR spectrum of commercially available 2PLP PDMS based ink IP-PDMS (Nanoscribe GmbH & Co. KG)*

1. **Raman spectra and calculation of acrylate conversion**

**^
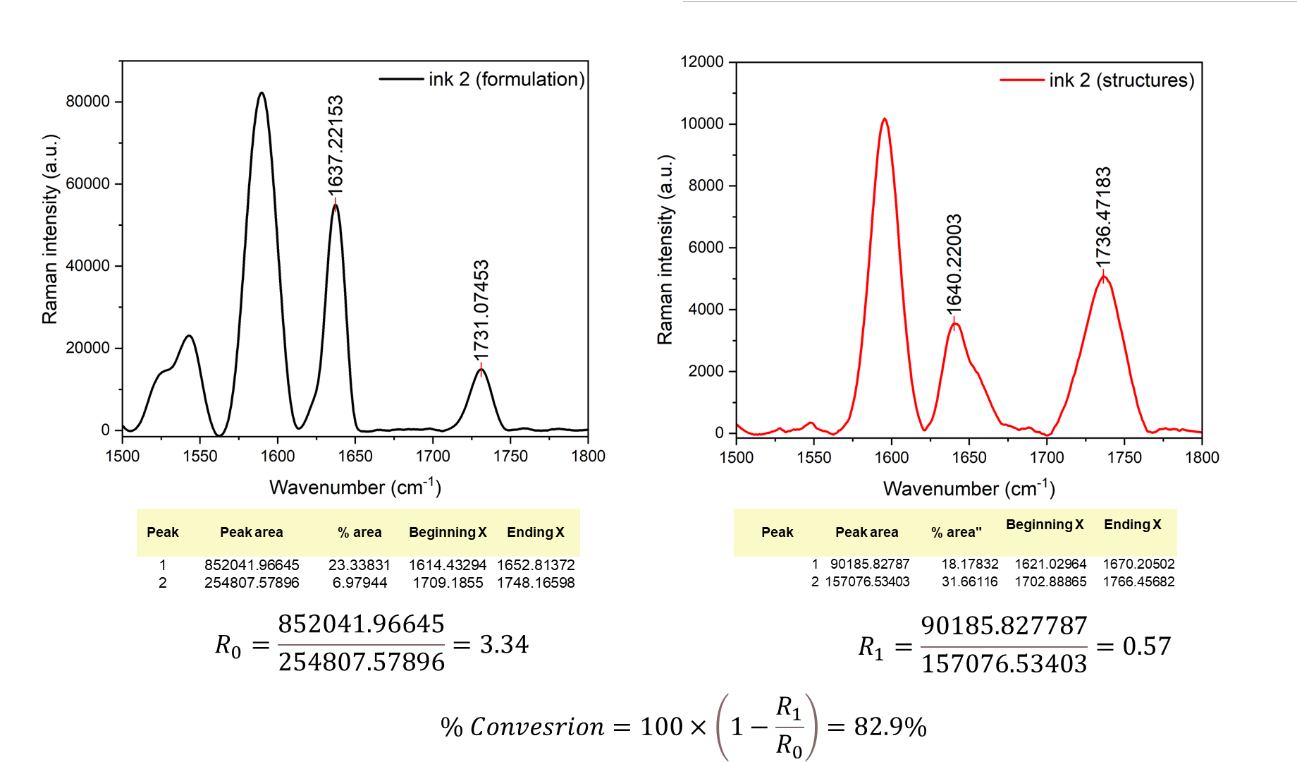
^**

***Figure S6.*** *Raman spectra of ink 2 formulation (left) and ink 2 printed structures (right) and calculation of acrylate conversion.*

**^
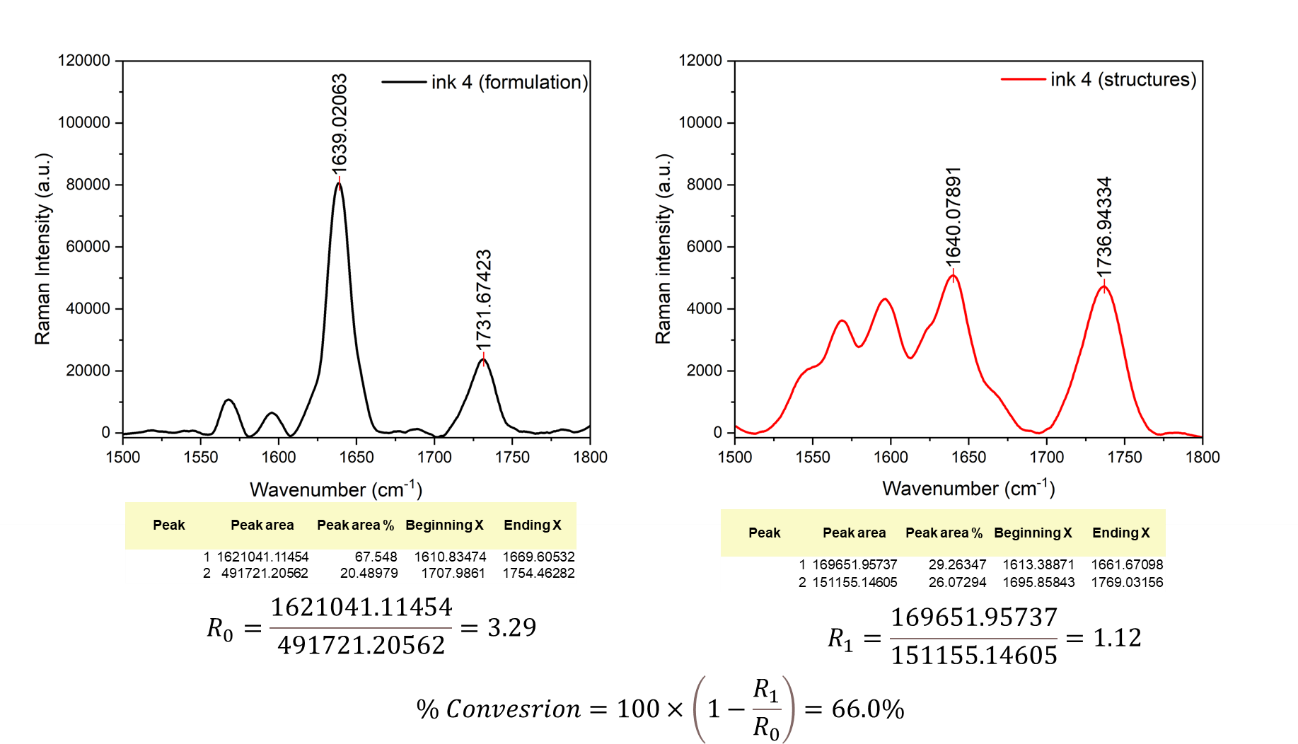
^**

***Figure S7.*** *Raman spectra of ink 4 formulation (left) and ink 4 printed structures (right) and calculation of acrylate conversion.*

**^
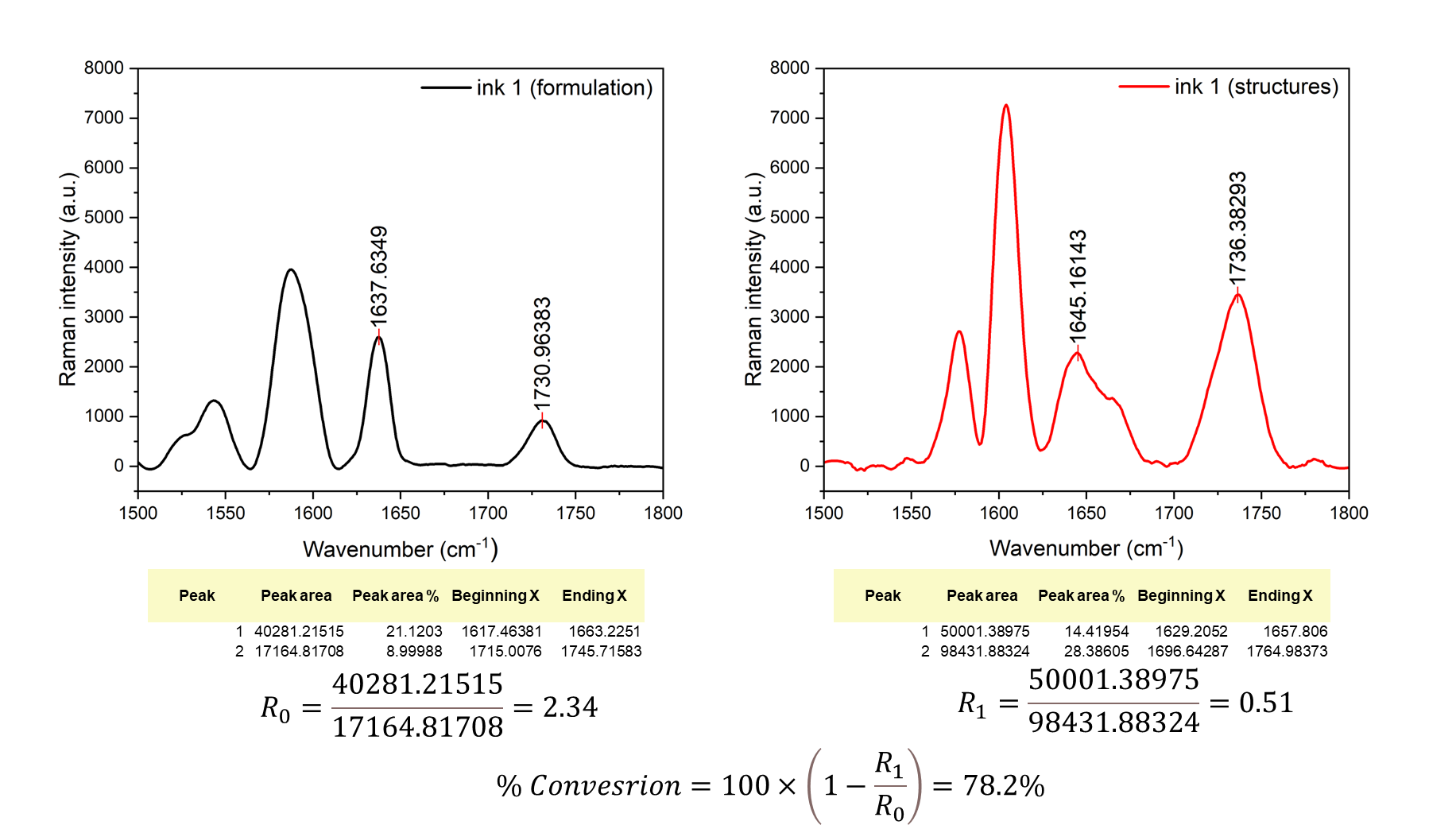
^**

***Figure S8.*** *Raman spectra of ink 1 formulation (left) and ink 1 printed structures (right) and calculation of acrylate conversion.*

**^
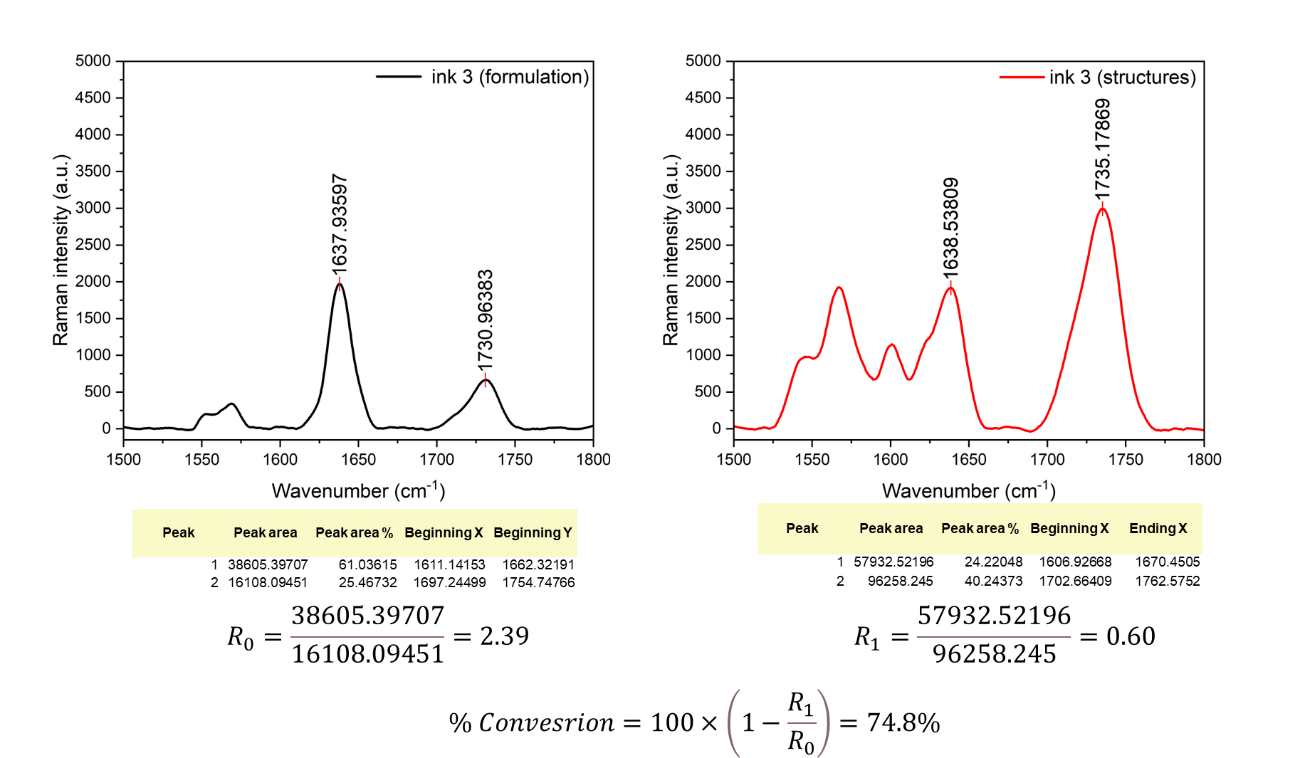
^**

***Figure S9.*** *Raman spectra of ink 3 formulation (left) and ink 3 printed structures (right) and calculation of acrylate conversion.*

1. **Printability evaluation**


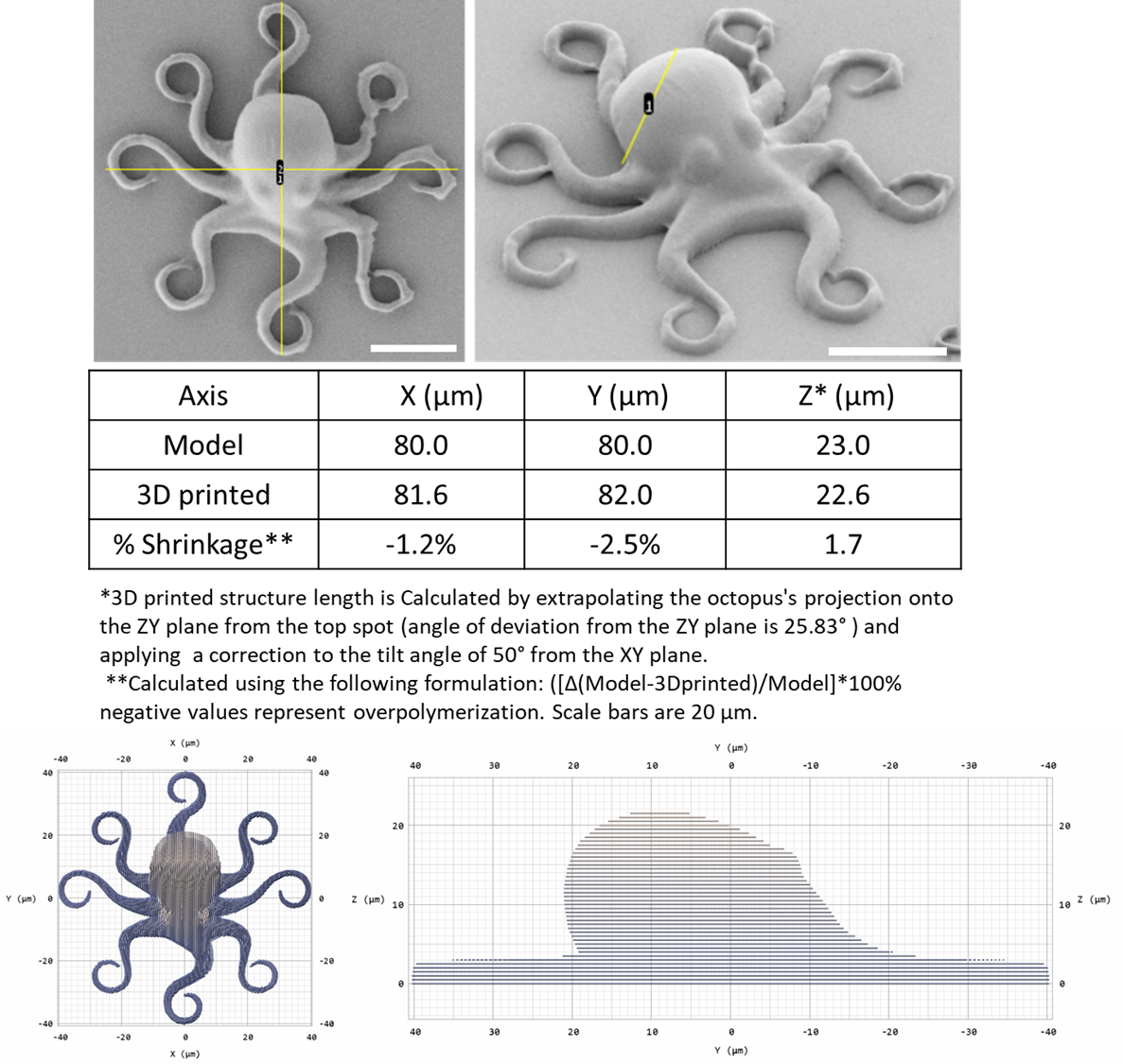


***Figure S10.*** *Printability evaluation of octopus printed using ink 1. Top: SEM Images and measurement marks for xy and z axis. Bottom: STL model file with designed dimensions.*


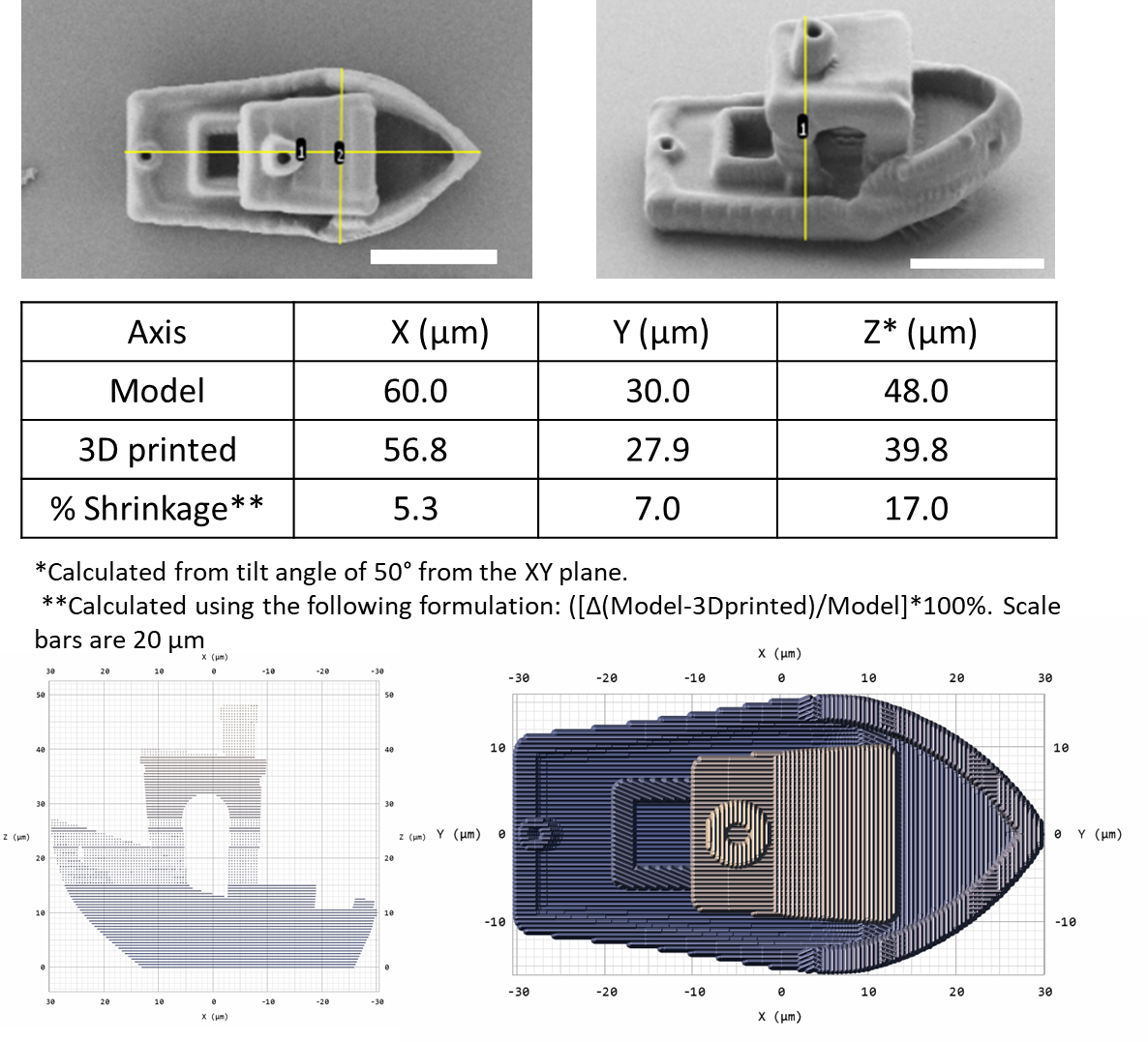


***Figure S11****. Printability evaluation of 3DBenchy printed using ink 2. Top: SEM Images and measurement marks for xy and z axis. Bottom: STL model file with designed dimensions.*

1. **Development an improved nanoindentation protocol for 3D printed elastomers at the microscale**


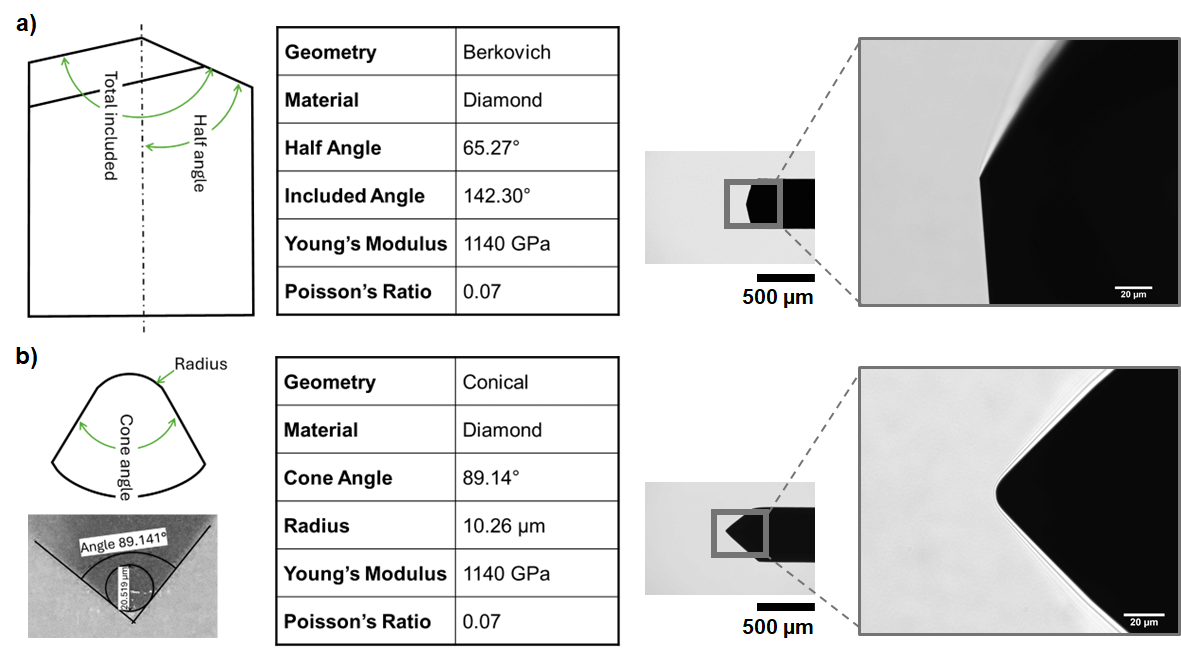


**Figure S12.** Detailed specifications and microscope images of the probe tips used in this study: (a) Berkovich and (b) conospherical tip.


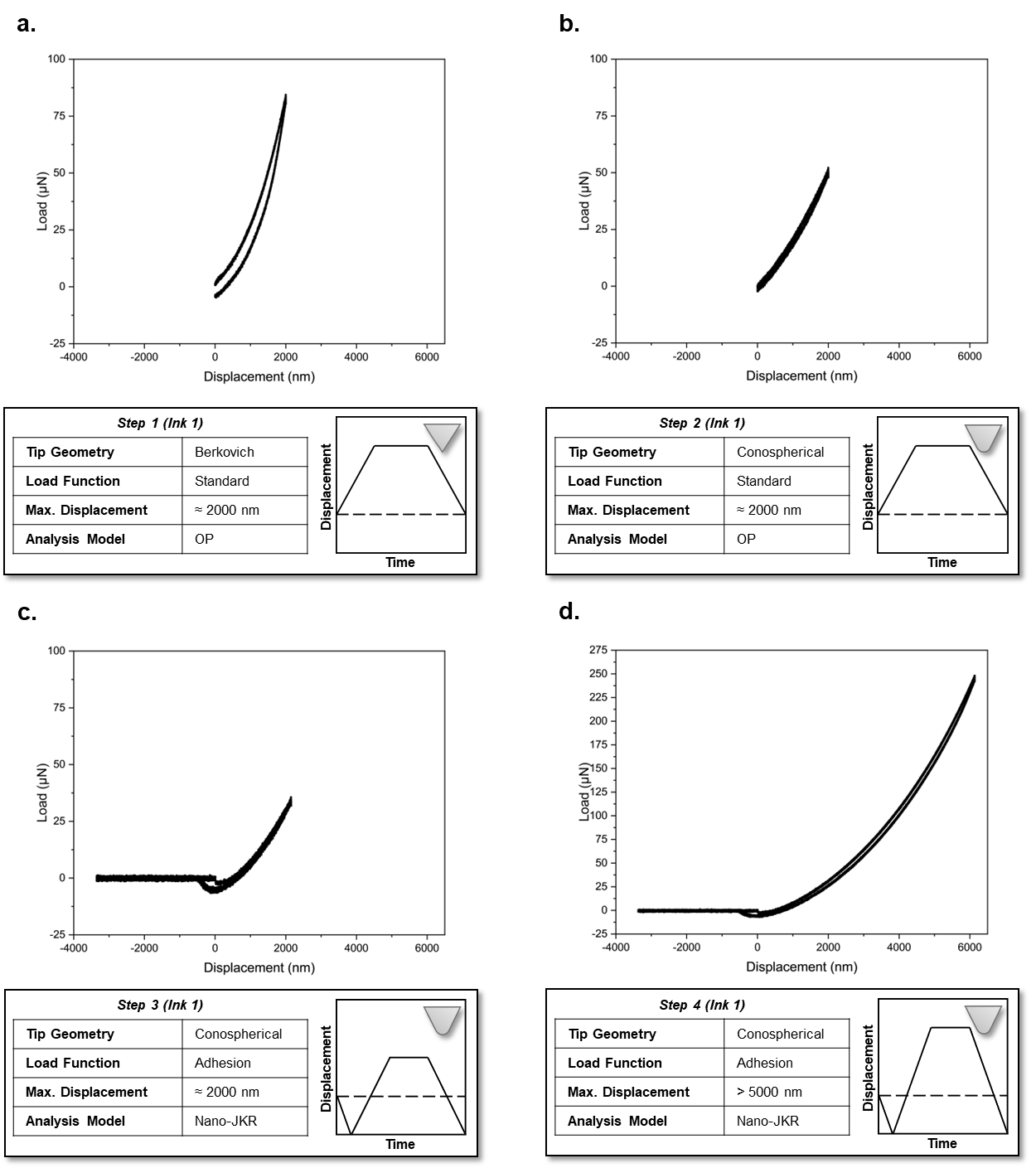


***Figure S13.*** *Exemplary load-displacement response of ink 1 during the stepwise optimization of the nanoindentation protocol. (a) Step 1: ‘Standard’ displacement-controlled trapezoidal displacement profile using a Berkovich tip and the OP model for analysis. (b) Step 2: ‘Standard’ displacement-controlled trapezoidal displacement profile using a conospherical tip (tip diameter = 10.26 µm) and the OP model for analysis. (c) Step 3: Adhesion-adapted displaced controlled displacement profile with lift-off segments at the start and end of the indent and the nano-JKR model for analysis. (d) Step 4: Deeper adhesion-adapted indents to find the optimal indentation depth.*

***Table S1.*** *Reduced moduli E_r_ measured via nanoindentation while performing the stepwise optimization of the nanoindentation protocol for tacky 3D printed elastomers at the microscale (ink* ***1*** *and ink* ***4****). All measurements were performed in triplicates, and the results are shown as mean ± SD.*

| **Ink** | **Step 1 [MPa]^a^** | **Step 2 [MPa]^b^** | **Step 3 [MPa]^c^** | **Step 4 [MPa]^d^** |
| --- | --- | --- | --- | --- |
| ***Ink 1*** | 10.23 ± 0.44 | 4.83 ± 0.03 | 2.07 ± 0.05 | 2.70 ± 0.02 |
| ***Ink 3*** | 6.88 ± 1.95 | 2.42 ± 0.06 | 0.90 ± 0.04 | 1.30 ± 0.02 |

*^a^Step 1: ‘Standard’ displacement-controlled trapezoidal displacement profile (peak displacement = 2000 nm) using a Berkovich tip and the OP model for analysis. ^b^Step 2: ‘Standard’ displacement-controlled trapezoidal displacement profile (peak displacement = 2000 nm) using a conospherical tip (tip diameter = 10.26 µm) and the OP model for analysis. ^c^Step 3: Adhesion-adapted displaced controlled displacement profile with lift-off segments at the start and end of the indent (peak displacement = 2000 nm, lift-off displacement = 4000 nm) using a conospherical tip (tip diameter = 10.26 µm) and the nano-JKR model for analysis. ^d^Step 4: ‘Deep’ adhesion-adapted displaced controlled displacement profile with lift-off segments at the start and end of the indent (peak displacement > 5000 nm, lift-off displacement = 4000 nm) using a conospherical tip (tip diameter = 10.26 µm) and the nano-JKR model for analysis.*

*
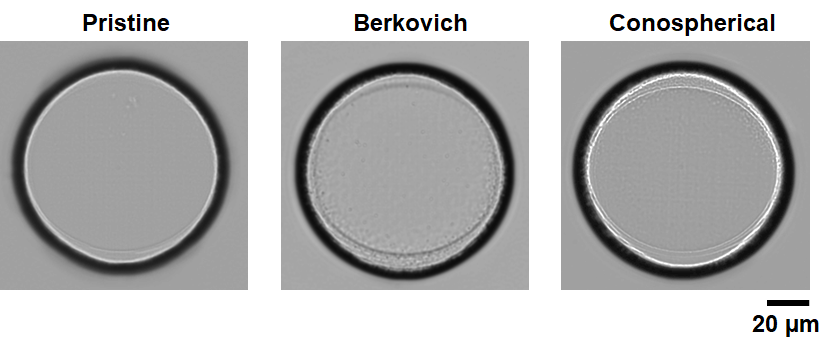
*

***Figure S14.*** *Comparison of three 3D printed pillars printed using ink 1, before and post nanoindentation showing (left) pristine surface, (center) post-indentation with a Berkovich tip and (right) post-indentation with a conospherical tip.*

*
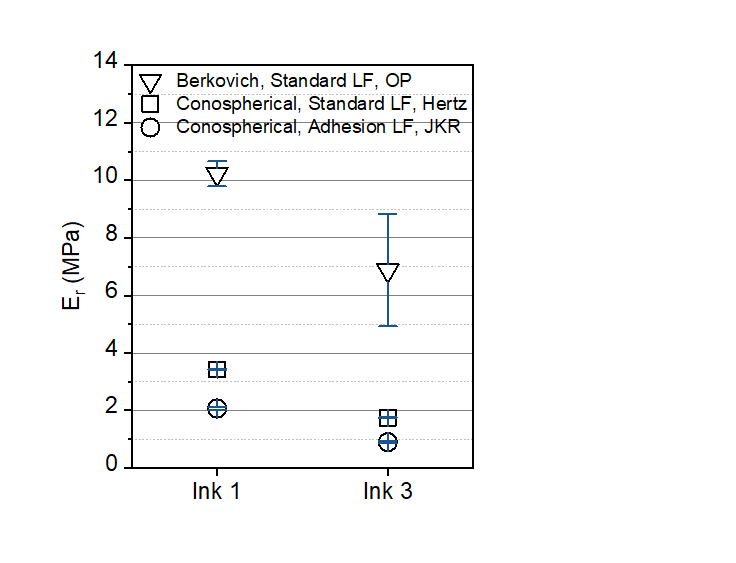
*

***Figure S15.*** *Comparison of the nanoindentation results of the ‘standard’ protocol employing a Berkovich tip with a trapezoidal displacement profile (DP) and the Oliver-Pharr (OP) model, the ‘standard’ trapezoidal DP using a conospherical tip (tip radius R = 10.26 µm) and the Hertzian model for analysis, and the adhesion-adapted DP and the nano-Johnson-Kendall-Roberts (nano-JKR) model.*

***Table S2.*** *Reduced moduli E_r_ measured via the optimized protocol (this work) compared to the moduli obtained via the ‘standard’ protocol. All measurements were performed in triplicates, and the results are shown as mean ± SD.*

| **Ink** | **This work (optimized) [MPa]^a^** | **Standard procedure [MPa]^b^** |
| --- | --- | --- |
| ***Ink 1*** | 2.07 ± 0.05 | 10.23 ± 0.44 |
| ***Ink 2*** | 2.80 ± 0.14 | 11.87 ± 0.09 |
| ***Ink 3*** | 0.90 ± 0.04 | 6.88 ± 1.95 |
| ***Ink 4*** | 1.31 ± 0.03 | 5.06 ± 0.35 |
| ***IP-PDMS*** | 2.46 ± 0.13 | 21.99 ± 0.59 |

*^a^This work: Optimized adhesion-adapted displaced controlled displacement profile with lift-off segments at the start and end of the indent (peak displacement = 2000 nm, lift-off displacement = 4000 nm) using a conospherical tip (tip diameter = 10.26 µm) and the nano-JKR model for analysis. ^b^Standard procedure: ‘Standard’ displacement-controlled trapezoidal displacement profile (peak displacement = 2000 nm) using a Berkovich tip and the OP model for analysis.*
